# Feasibility of the new copper pipe method for evaluating half‐value layer in computed tomography: A measurement and Monte Carlo simulation study

**DOI:** 10.1002/acm2.12780

**Published:** 2019-11-25

**Authors:** Rena Okubo, Kosuke Matsubara, Thunyarat Chusin, Tomoya Hibino, Yusuke Ito

**Affiliations:** ^1^ Department of Radiological Technology DAIYUKAI HEALTH SYSTEM Ichinomiya Japan; ^2^ Department of Quantum Medical Technology Faculty of Health Sciences Institute of Medical Pharmaceutical and Health Sciences Kanazawa University Kanazawa Japan; ^3^ Department of Quantum Medical Technology Graduate Course of Medical Science and Technology Division of Health Sciences Graduate School of Medical Science Kanazawa University Kanazawa Japan

**Keywords:** computed tomography, effective energy, half‐value layer, Monte Carlo simulation

## Abstract

This study aimed to verify the accuracy of half‐value layer (HVL) measured using the new copper pipe method with the CT ionization chamber while the X‐ray tube is rotating and to compare it with the conventional nonrotating method and Monte Carlo simulation method based on the actual measurement and geometry of the new copper pipe method. HVL was measured while the X‐ray tube was rotating using a CT ionization chamber surrounded by copper pipe absorbers and located at the isocenter of the CT gantry. The exposure as the copper pipe thickness approached 0 mm was extrapolated from the attenuation curve to take the influence of scatter radiation into consideration. The results of the new copper pipe method were compared with those of the other two methods. Data were acquired using two different CT scanners on a single axial scan. The two one‐sided test (TOST) equivalent test yielded equivalence between HVLs derived from the new copper pipe and the nonrotating methods (*P* < 0.05) and those derived from the new copper pipe and the simulation methods (*P* < 0.05) at the equivalence margins of ± 0.03 mmCu. The mean absolute difference in HVL between the new copper pipe and conventional nonrotating methods was 0.01 ± 0.02 mmCu, which corresponded to an error of effective energy of (0.86 ± 1.66)%. The new copper pipe method can ensure that HVL of CT scanner can easily be evaluated using solely the CT ionization chamber and copper pipe absorbers without requiring service engineering mode.

## 1 INTRODUCTION

Half‐value layer (HVL) is a practical indicator that is used for describing polyenergetic radiation. HVL is used in various X‐ray imaging techniques, such as fluoroscopy, radiography, and mammography, to assess the beam quality. However, the measurement of HVL in computed tomography (CT) is complicated because the X‐ray tube has to be accurately placed using the unit’s service engineering mode. For increased test validity, several researchers have investigated different methods to measure HVL while the X‐ray tube is rotating.^1^, ^2^, ^3^, ^4^, ^5^

Previous studies have investigated noninvasive methods to measure HVL while the X‐ray tube is rotating; however, these studies were limited by the difficulty in creating the necessary equipment and in accurately placing the CT ionization chamber. For example, the ring method^1^ does not require parking the X‐ray tube to measure HVL; instead, HVL is measured while the tube is rotating by placing the CT ionization chamber at the center of the gantry, and surrounding it with aluminum rings. Thus, each projection of the X‐ray beam is filtered by an identical amount of aluminum, and this method requires custom‐built acrylic stands to support the aluminum rings. The localization method^1^ uses the CT’s localizer mode to measure HVLs. This method is similar to the conventional nonrotating method; however, it requires a special support mechanism for the CT ionization chamber to prevent it from moving when the couch moves. The lead‐covered case method^2^ can also measure HVL without using the service engineering mode. The box, made of lead plates, blocks out the X‐ray beam, except for an aperture in the box. Attenuators, such as aluminum or copper filters, are placed on this aperture to measure HVL. The lead box is easy to manufacture; however, its bulkiness and the placement of the CT ionization chamber prove to be the drawbacks associated with this method.

The copper pipe method,^3^ which is one of the HVL evaluation methods, uses general‐purpose ionization chamber and several thicknesses of copper pipes to determine HVL. The copper pipes were fabricated to attach general purpose ionization chamber (6 cc thimble). The diameter and length of the copper pipes were 20 and 40 mm respectively. An ionization chamber was covered with them, exposure was measured, and attenuation curves were derived. The attenuation curves of semilogarithmic plots were approximated by cubic function from 0 to 0.3 mm thickness of copper pipes, and quadratic function from 0.3 to 0.6 mm. HVLs were derived as the thickness of copper required to attenuate one‐half of the incident radiation by using these attenuation curves. This method does not need special support for the CT ionization chamber; instead, the chamber can be placed anywhere within a field of view. However, this method has some unsolved problems. First, the method uses a general‐purpose ionization chamber, which is not commonly used for dose measurement in CT. Second, choosing the correct approximation function depends on the thickness of the copper pipe. For example, from 0 to 0.3 mm, a cubic function is used, whereas from 0.3 to 0.6 mm, a quadratic function is used. Third, the error in effective energy between the copper pipe method and the conventional nonrotating method is relatively large, even after applying a correction factor (1.81 ± 1.38%).

The present study aimed to solve the abovementioned problems and verify the accuracy of the new copper pipe method in measuring HVLs in CT scanners while the X‐ray tube is rotating. The new method was compared with the conventional nonrotating method and a simulation method using the geometry of the new copper pipe method.

## 2 MATERIALS AND METHODS

### 2.1 CT scanners and dosimeter

HVLs were obtained from a 16‐channel (SOMATOM Emotion; Siemens Healthineers, Erlangen, Germany) and 64‐channel (LightSpeed VCT; GE Healthcare, Milwaukee, WI, USA) multidetector row CT (MDCT) scanners. Exposure was measured using the CT ionization chamber (model 10X6‐3 CTDI chamber; Radcal, Monrovia, CA, USA) and an electrometer (model 2026C electrometer; Radcal).

### 2.2 New copper pipe method

**Figure 1:**
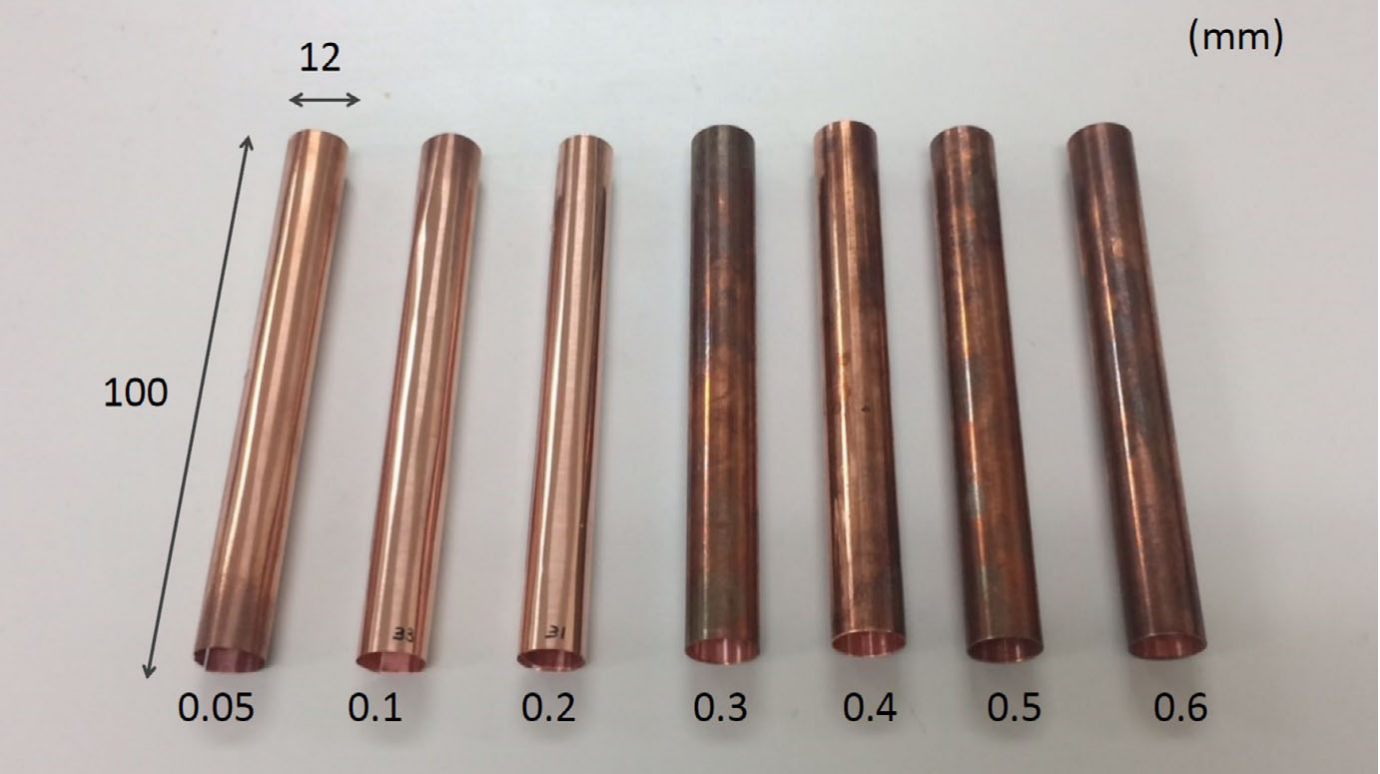
Fabricated copper pipes for measuring the half‐value layer in a CT scanner.

Figure 1 shows the fabricated copper pipes made of copper (C1100 or C1220), with thicknesses of 0.05, 0.1, 0.2, 0.3, 0.4, 0.5, and 0.6 mm. These materials were chosen for their capability of being shaped and high purity (>99.9%). The length and diameter of these pipes were 100 and 12 mm respectively.

**Figure 2:**
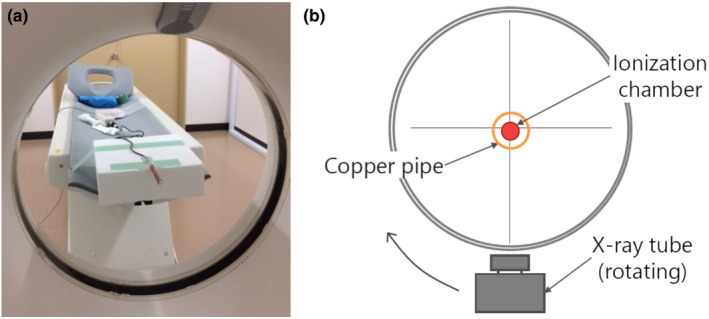
The experimental setup for the new copper pipe method for measuring half‐value layer (HVL). (a) A position of covered ionization chamber at the center of the gantry. (b) A schema of the new copper pipe method.

Figure 2 shows the experimental setup of the new copper pipe method. Briefly, the CT ionization chamber was placed at the center of the gantry and covered by each thickness of the copper pipe. Exposure values were recorded using CT scanners with the following single axial scan parameters: tube voltages of 80, 110, and 130 kV and a detector configuration of 8 × 1.2 mm for the 16‐channel MDCT scanner and tube voltages of 80, 100, 120, and 140 kV and a detector configuration of 4 × 0.625 mm for the 64‐channel MDCT scanner. A tube current of 50 mA, X‐ray tube rotation time of 1.0 s per rotation, and a small focal spot size were selected for both CT scanners. The beam width differed between the two CT scanners because of their different detector configurations. Each series of data were log‐transformed, and attenuation curves were derived using the least‐squares method. Exposures were measured at least thrice and averaged. The exposures as the copper pipe thickness approached 0 mm ($I_{0}^{\prime }$) were extrapolated from the approximate curves to take the effect of the scattered radiation into consideration.

The ratio of $I_{0}^{\prime }$ and the exposure at HVL is shown in the following equation:

(1) $$\begin{array}{c} \frac{I_{\text{HVL}}}{I_{0}^{\prime }}=\frac{1}{2} \end{array}$$

where $I_{\mathrm{HVL}}$ represents exposure at HVL. When taking the logarithm of Eq. (1),

(2) $$\begin{array}{c} \log\frac{I_{\text{HVL}}}{I_{0}^{\prime }}=\log\frac{1}{2} \end{array}$$

The exposure at HVL will be described by following formula,

(3) $$\begin{array}{c} \log\;\;I_{\text{HVL}}=\log\;I_{0}^{\prime }-\log\;2 \end{array}$$

HVL was determined by using Eq. (3) and the goal‐seek feature in the what‐if analysis tool of Microsoft Excel 2019 (Microsoft Corporation, Redmond, WA, USA).

### 2.3 Conventional nonrotating method

**Figure 3:**
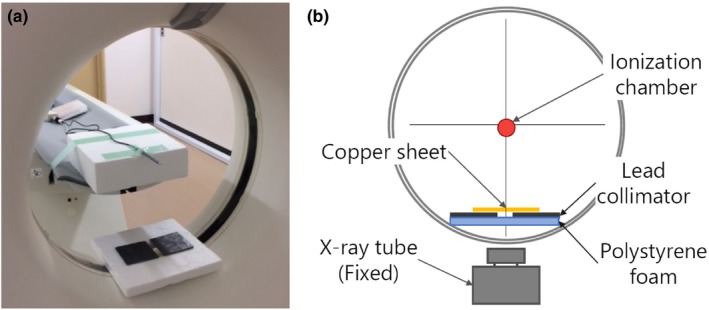
The experimental setup for the conventional nonrotating method. (a) A position of the ionization chamber and the lead collimator at the gantry. (b) A schema of the nonrotating conventional method.

Figure 3 shows the experimental setup of the conventional nonrotating method. Briefly, the scan parameters were kept the same as the new copper pipe method, except that the X‐ray tube was parked at the bottom of the gantry using the service engineering mode. Exposure time was set to 1.0 s. The ionization chamber was placed at the center of the gantry. In this method, 10 × 10‐cm pieces of copper sheet with thicknesses of 0.1, 0.2, 0.3, 0.4, and 0.5 mm were used to attenuate the X‐ray beam. These filters were placed on a lead collimator above the lower surface of the gantry. Exposure was measured at least thrice and averaged. The equivalence of HVLs derived from the copper pipe and the nonrotating methods were tested using two one‐sided test (TOST) with a margin of 0.03 mmCu.

### 2.4 Monte Carlo simulation based on the new copper pipe method

The copper pipe method was simulated using the Particle and Heavy Ion Transport Code System (PHITS) ver. 3.00.^6^ The free software X‐Tucker‐3, which calculates X‐ray energy spectra by using a corrected Tucker's formula,^7^ was used for estimating the X‐ray energy spectrum of the original CT X‐ray source by using tube voltage, target angle, and the thickness of inherent filters. The 80, 110, and 130 kV spectra were calculated for a 16‐channel MDCT scanner, and those of 80, 100, 120, and 140 kV spectra were obtained for a 64‐channel MDCT scanner. Attenuation curves obtained from the conventional nonrotating method were used to determine the shape of the spectrum. Because the material and thickness of the inherent filter were not described, the material of the filter was assumed to be aluminum and the filter thickness was determined based on an identical attenuation curve obtained from a conventional nonrotating method. In this study, exposure was measured only at the center of the gantry; thus, the thickness of inherent filter included the thickness of bowtie filter at the center.

The simulation geometry was defined based on the actual measurements of the copper pipe method. A source‐to‐isocenter distance and X‐ray beam angle were chosen based on each CT scanner specification. A fan‐shaped beam was defined using a default cone‐beam and a pair of collimators. Because the couch was not in the beam path, it was not defined in the simulation. The calculated spectrum was imported into PHITS, and particle transportation was simulated. Batch and history numbers were set to minimize the relative error to <1.0%. Equivalence of the copper pipe and the simulation methods were analyzed using TOST equivalence test with a margin of 0.03 mmCu to determine statistical significance. Using the linear attenuation coefficient of copper, HVLs were converted to effective energies.^8^

## 3 RESULTS

**Figure 4:**
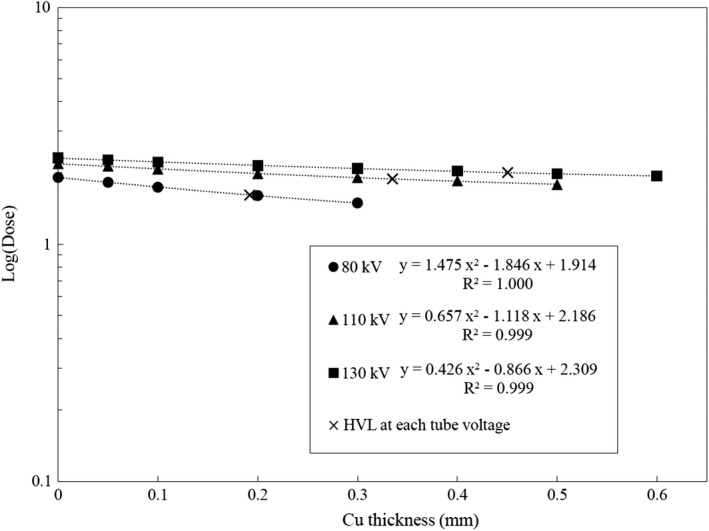
Attenuation curves for 16‐channel multidetector row CT (MDCT) derived from the new copper pipe method.

**Figure 5:**
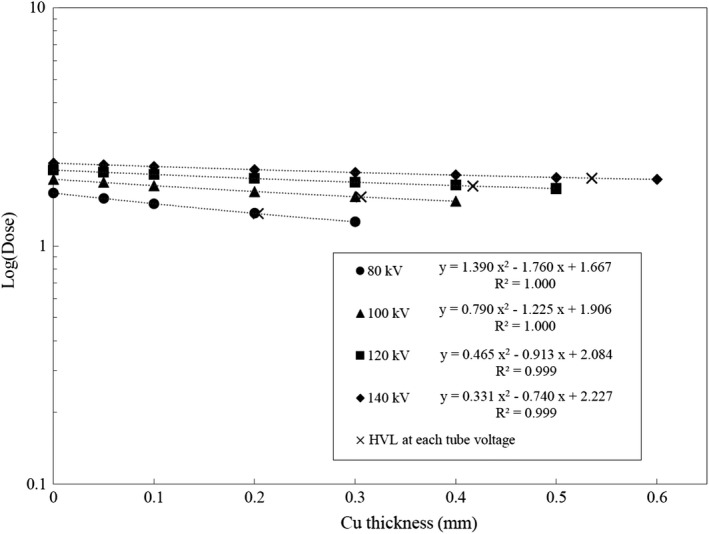
Attenuation curves for 64‐channel multidetector row CT (MDCT) derived from the new copper pipe method.

Attenuation curves derived from the new copper pipe method are shown in Fig. 4 for the 16‐channel MDCT and Fig. 5 for the 64‐channel MDCT. HVL at each tube voltage is shown as x mark on each attenuation curve. In this method, HVL was derived using quadratic function for the entire dataset because the polyenergetic photon beam was exposed to the attenuator and the CT ionization chamber. Because the attenuation curve did not follow exponential function, the quadratic function was used. The linear coefficient of determination for each attenuation curve was approximately 0.99. This trend was observed both in 16‐channel and 64‐channel MDCTs for all tube voltages. The standard deviation of each data point was <0.01.

Table 1 presents the results of HVLs measured using the conventional nonrotating, copper pipe, and simulation methods. The TOST procedure yielded equivalence between HVLs derived by the copper pipe and the nonrotating methods (*P* < 0.05) at the predefined equivalence margins of ± 0.03 mmCu. Systematic overestimation or underestimation of HVLs was not observed. The absolute error varied from −0.02 to 0.04 mmCu, which corresponds to a difference in effective energy of −0.77 to 1.84 keV. The mean absolute difference in HVLs measured using the new copper pipe and conventional nonrotating methods was 0.01 ± 0.02 mmCu. The relative error in effective energy was (0.89 ± 1.66)% for the new copper pipe method.

**Table 1:** Half‐value layer (HVL) measured by the three methods and expressed as both HVL and effective energy.

Scanner	Tube voltage (kV)	HVL (mmCu)			Effective energy (keV)		
		NR	CP‐M	CP‐S	NR	CP‐M	CP‐S
16‐channel MDCT	80	0.19	0.19	0.20	42.9	43.1	43.6
	110	0.31	0.34	0.32	51.1	52.8	51.9
	130	0.41	0.45	0.43	56.8	58.6	57.8
64‐channel MDCT	80	0.21	0.20	0.23	44.4	44.0	45.7
	100	0.30	0.31	0.33	50.6	51.0	52.3
	120	0.41	0.42	0.41	57.0	57.2	56.8
	140	0.55	0.54	0.57	63.4	62.6	64.0

NR, CP‐M, and CP‐S, for Nonrotating, copper pipe‐measurement, and copper pipe‐simulation.

The TOST procedure yielded equivalence between HVLs derived from the copper pipe and the simulation methods (*P* < 0.05) at the predefined equivalence margins of ± 0.03 mmCu, and no systematic overestimation or underestimation was observed. The absolute error varied from −0.02 to 0.03 mmCu in HVLs, which corresponds to a difference of −1.67 to 0.89 keV in effective energy. The average error estimated between the new copper pipe and simulation methods was −0.01 ± 0.02 mmCu. Thus, the data indicate that HVLs measured using the new copper pipe method agreed with both the conventional nonrotating method and the simulation method based on the new copper pipe method.

## 4 DISCUSSION

This study investigated the accuracy of the new copper pipe method, which can measure HVLs in a CT scanner while the X‐ray tube is rotating. This method was compared to the conventional nonrotating method and a simulation method based on the new copper pipe method. The result showed that the HVLs derived from the copper pipe method were equivalent with the conventional nonrotating and the simulation methods with a margin of 0.03 mmCu. Even considering an equivalent margin of 0.03, the corresponding difference in effective energy is approximately 2 keV. In addition, the actual difference in HVLs derived from the new copper pipe and the nonrotating method was less than 0.03 mmCu; thus, we believe that this difference was negligible.

Previous studies have investigated several methods to measure HVLs while the X‐ray tube is rotating. However, most of these methods require new, complicated equipment to support the attenuators and CT ionization chamber or CT chamber with special functions, such as a real‐time dosimeter.^1^, ^2^, ^3^, ^4^, ^5^ Though the copper pipes were developed by ourselves and they are not available in the market, the new copper pipe method is unique because it can measure HVLs using the existing CT ionization chamber without using complicated equipment to support them.

Comparing the former copper pipe method, HVL was consistently calculated using the new copper pipe method. In the former copper pipe method, different approximation functions were used based on the thicknesses of the copper pipe. In the new copper pipe method, HVL was measured using a quadratic function for the whole dataset. The limitation of the former copper pipe method can be attributed to the length and the diameter of the copper pipe, which is not large enough to remove scattered radiation from the X‐ray beam. On the other hand, the length and the diameter of the new copper pipe are longer and smaller than those of the former copper pipe. Because of this, scattered radiation appears to be removed by the longer copper pipe. The new copper pipe seems to contribute to minimize the amount of scattered radiation measured by the ionization chamber.

This study also demonstrated that HVLs measured using the new copper pipe method were equivalent to HVLs derived using the conventional nonrotating method. According to the present study, the relative error in effective energy was approximately 0.89% for the new copper pipe method. Iida et al.^3^ showed that the relative error in effective energy was 1.81% for the former copper pipe method, which was larger than that for the new copper pipe method. Moreover, the accuracy of the new copper pipe method was verified using the simulation method based on the geometry of new copper pipe method. The difference between HVLs measured by these methods were also equivalent. Notably, the average error estimated between the actual measurement and simulation methods based on the new copper pipe method was relatively small. This result implies that the accuracy of the new copper pipe method can be shown by not only comparing nonrotating conventional method but also simulation method based on the new copper pipe method. The new copper pipe method also has advantages in data collection and analysis over the former copper pipe method.

The new copper pipe method has several future perspectives. For over 10 years, some medical institutions have used dual energy CT (DECT) for image processing, such as monoenergetic image reconstruction, to decrease beam‐hardening effects and bone removal in CT angiography.^9^ However, there are few methods to evaluate the X‐ray energy in DECT.^10^ Understanding HVL as a basic physical characteristic of DECT is of importance. The copper pipe method possibly applies to the split‐beam DECT. By applying this method, it could be possible to obtain average HVL of two type of energy spectra. This method is considered to contribute to derive the mass energy coefficient ratio and calculate organ dose when this type of the scanners are used.^10^, ^11^ There is another perspective, the copper pipe method may allow the estimation of the spectrum inside the dosimetry phantom. The diameter of the CT ionization chamber is 9 mm,^12^ and the chamber ports diameter of the CTDI phantom is 13.1 mm.^13^ Therefore, it is possible to insert the CT chamber covered with copper pipe into the CTDI phantom. By putting CT ionization chamber covered by copper pipe absorbers, HVLs inside the phantom can be evaluated. We believe measuring HVLs inside the phantom can provide new aspect to understand the dose estimation for patients.

Some limitations of this study must be noted. First, only two CT scanners were used to verify the accuracy of HVL measured using the copper pipe method. Although the average error between the conventional nonrotating and copper pipe methods was approximately 0.01 mmCu in this study, it may be different in other CT scanners. Secondly, aluminum HVL was not discussed in this study. Although some reports suggested that the aluminum HVL should be used on diagnostic field,^14^ copper is also appropriate as the attenuator for measuring HVL in the energy range of CT scan.^15^ Thirdly, the present study did not explain the mechanism of the scattered radiation correction and the effect of the beam hardening enough. However, the differences between HVLs derived from copper pipe method and those derived from the conventional method were not significant. In a future study, the change in spectrum due to beam hardening should be investigated to determine a better correction method. Lastly, the X‐ray energy spectrum for the simulation method was not directly measured. Instead, the X‐ray energy spectrum was calculated from the tube voltage, target angle, and thickness of inherent filter. Previous study showed that measured doses and simulated doses using spectra calculated by Tucker’s formula were agreed well,^16^ thus we believe that defined spectrum was sufficiently precise to simulate measured exposure.

## 5 CONCLUSION

The study demonstrated the ease and inexpensiveness of the new copper pipe method that also improved on the accuracy of the former method. It was observed that the new copper pipe method can measure HVL while the X‐ray tube is rotating as efficiently as the conventional nonrotating method. It required neither the use of service engineering mode nor any special support mechanism. This method can ensure that HVL of CT scanner can easily be evaluated with minimal equipment, and it is applicable in clinical setting for quality assurance of CT scanner and dose estimation for patients.

## CONFLICT OF INTEREST

The authors declare no conflict of interest.

## References

[1] Kruger RL , McCollough CH , Zink FE . Measurement of half‐value layer in x‐ray CT: a comparison of two noninvasive techniques. Med Phys. 2000;27:1915–1919. 10984237 10.1118/1.1287440

[2] Matsubara K , Ichikawa K , Murasaki Y , Hirosawa A , Koshida K . Accuracy of measuring half‐ and quarter‐value layers and appropriate aperture width of a convenient method using a lead‐covered case in X‐ray computed tomography. J Appl Clin Med Phys. 2014;15:309–316. 10.1120/jacmp.v15i1.4602 PMC5711220 24423861

[3] Iida H , Noto K , Mitsui W , Takata T , Yamamoto T , Matsubara K . New method of measuring effective energy using copper‐pipe absorbers in X‐ray CT. Nihon Hoshasen Gijutsu Gakkai Zasshi. 2011:1183–1191. 21937842 10.6009/jjrt.67.1183

[4] Maia AF , Caldas LVE . A simple method for evaluation of half‐value layer variation in CT equipment. Phys Med Biol. 2006;51:1595–1601. 16510965 10.1088/0031-9155/51/6/016

[5] McKenney SE , Anthony Seibert J , Burkett GW , et al. Real‐time dosimeter employed to evaluate the half‐value layer in CT. Phys Med Biol. 2014;59:363–377. 24351935 10.1088/0031-9155/59/2/363 PMC4369799

[6] Sato T , Iwamoto Y , Hashimoto S , et al. Features of Particle and Heavy Ion Transport code System (PHITS) version 3.02. J Nucl Sci Technol. 2018;3131:1–7.

[7] Tucker DM , Barnes GT , Chakraborty DP . Semiempirical model for generating tungsten target x‐ray spectra. Med Phys. 1991;18:211–218. 2046607 10.1118/1.596709

[8] Hubbell JH , Seltzer SM .Tables of x‐ray mass attenuation coefficients and mass energy‐absorption coefficients 1 keV to 20 MeV for elements Z = 1 to 92 and 48 additional substances of dosimetric interest. https://www.nist.gov/publications/tables-x-ray-mass-attenuation-coefficients-and-mass-energy-absorption-coefficients-1-0. Published 1995. Accessed January 6, 2018.

[9] McCollough CH , Leng S , Yu L , Fletcher JG . Dual‐ and multi‐energy CT: principles, technical approaches, and clinical applications. Radiology. 2015;276:637–653. 26302388 10.1148/radiol.2015142631 PMC4557396

[10] Matsubara K , Nagata H , Okubo R , Takata T , Kobayashi M . Method for determining the half‐value layer in computed tomography scans using a real‐time dosimeter: application to dual‐source dual‐energy acquisition. Phys Medica. 2017;44:227–231. 10.1016/j.ejmp.2017.10.020 29111386

[11] Almeida IP , Schyns LEJR , Ollers MC . Dual‐energy CT quantitative imaging: a comparison study between twin‐beam and dual‐source CT scanners. Med Phys. 2017;44:171–179. 28070917 10.1002/mp.12000

[12] Radcal Corporation . Specifications of 10X6‐3CT The Chamber for Computed Tomography Dose Index (CTDI). http://radcal.com/rdclwp/wp-content/uploads/2016/10/radcal-10X6-3CT-chamber-spec-sheet.pdf. Published 2011.

[13] GAMMEX A SUN NUCLEAR COMPANY . CTDI phantoms. https://www.sunnuclear.com/documents/datasheets/ctdi_phantoms.pdf. Published 2015.

[14] International Atomic Energy Agency (IAEA) . Dosimetry in diagnostic radiology: an international code of practice. IAEA Technical Reports Series No. 457 Vienna, Austria: IAEA; 2007.

[15] International Commission on Radiation Units and Measurements (ICRU) . Physical aspects of irradiation. ICRU report 10b. 1962.

[16] Fujii K , Nomura K , Muramatsu Y , Obara S , Akahane K , Kusumoto M . Organ dose evaluation based on monte carlo simulation for CT examinations using tube current. Radiat Prot Dosim. 2017;28;174(3):387–394. 10.1093/rpd/ncw144 27342451

